# Prediction of Drug-Induced Hyperbilirubinemia by In Vitro Testing

**DOI:** 10.3390/pharmaceutics12080755

**Published:** 2020-08-11

**Authors:** Péter Tátrai, Péter Krajcsi

**Affiliations:** 1 Solvo Biotechnology, Science Park, Building B1, 4-20 Irinyi József utca, H-1117 Budapest, Hungary; tatrai@solvo.com; 2 Faculty of Health Sciences, Semmelweis University, H-1085 Budapest, Hungary; 3 Faculty of Information Technology and Bionics, Péter Pázmány Catholic University, H-1083 Budapest, Hungary

**Keywords:** OATP1B1, UGT1A1, hyperbilirubinemia, in vitro, risk assessment

## Abstract

Bilirubin, the end product of heme catabolism, is produced continuously in the body and may reach toxic levels if accumulates in the serum and tissues; therefore, a highly efficient mechanism evolved for its disposition. Normally, unconjugated bilirubin enters hepatocytes through the uptake transporters organic anion transporting polypeptide (OATP) 1B1 and 1B3, undergoes glucuronidation by the Phase II enzyme UDP glucuronosyltransferase 1A1 (UGT1A1), and conjugated forms are excreted into the bile by the canalicular export pump multidrug resistance protein 2 (MRP2). Any remaining conjugated bilirubin is transported back to the blood by MRP3 and passed on for uptake and excretion by downstream hepatocytes or the kidney. The bile salt export pump BSEP as the main motor of bile flow is indirectly involved in bilirubin disposition. Genetic mutations and xenobiotics that interfere with this machinery may impede bilirubin disposition and cause hyperbilirubinemia. Several pharmaceutical compounds are known to cause hyperbilirubinemia via inhibition of OATP1Bs, UGT1A1, or BSEP. Herein we briefly review the in vitro prediction methods that serve to identify drugs with a potential to induce hyperbilirubinemia. In vitro assays can be deployed early in drug development and may help to minimize late-stage attrition. Based on current evidence, drugs that behave as mono- or multispecific inhibitors of OATP1B1, UGT1A1, and BSEP in vitro are at risk of causing clinically significant hyperbilirubinemia. By integrating inhibition data from in vitro assays, drug serum concentrations, and clinical reports of hyperbilirubinemia, predictor cut-off values have been established and are provisionally suggested in this review. Further validation of in vitro readouts to clinical outcomes is expected to enhance the predictive power of these assays.

## 1. Bilirubin in Health

**Figure 1:**
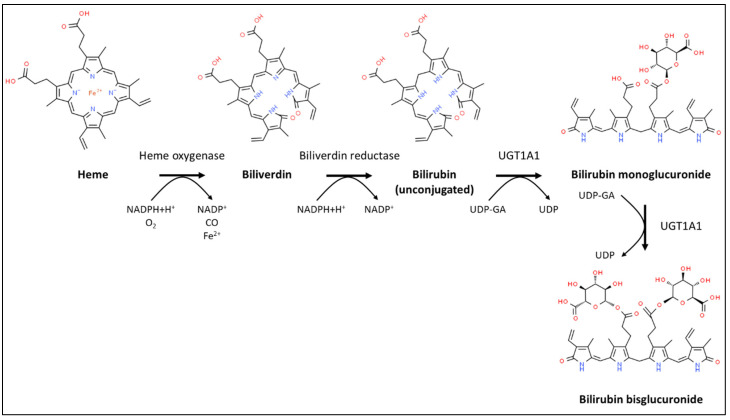
Schematic diagram of heme degradation and bilirubin conjugation.

Bilirubin, a linear tetrapyrrole, is the catabolic end product of the heme prosthetic group found in hemoglobin, myoglobin, and cytochrome P450 enzymes. Disintegrating red blood cells are the major source of bilirubin as approx. 80% of bilirubin is derived from the degradation of erythrocyte hemoglobin in the reticuloendothelial system [1]. Heme is first cleaved by the enzyme heme oxygenase to yield biliverdin that is subsequently reduced to bilirubin by biliverdin reductase [2]; finally, UDP-glucuronosyltransferase 1A1 (UGT1A1) sequentially attaches glucuronic acid moieties to bilirubin (Figure 1). Bilirubin mono- and bisglucuronides are collectively referred to as conjugated bilirubin.

Bilirubin can be present in three forms in the serum. Unconjugated bilirubin, also called indirect bilirubin, is poorly soluble and most of it is reversibly bound to albumin. The other two forms, collectively referred to as direct bilirubin, consist of free conjugated bilirubin and conjugated bilirubin covalently bound to albumin (also known as delta-bilirubin). Reference serum levels for adults are ≤25 µM for total bilirubin and ≤2 µM for conjugated bilirubin [3]. Unconjugated bilirubin is carried to the liver by systemic blood. Once taken up into hepatocytes, it undergoes glucuronidation and becomes excreted mostly into bile and to a lesser extent back to the blood.

## 2. Disruption of Bilirubin Homeostasis: Hyperbilirubinemia

Possible causes of hyperbilirubinemia include conditions with elevated bilirubin formation such as hemolytic anemia [4], ineffective erythropoiesis, or rhabdomyolysis [3]. The excess bilirubin in these cases is usually unconjugated and rarely exceeds 100 µM. Impaired elimination of bilirubin in liver diseases due to genetic predisposition, hepatitis, cholestasis, or cirrhosis can also precipitate hyperbilirubinemia [5]. Conjugated bilirubin in the plasma always indicates a pathological process. In conjugated hyperbilirubinemia the conjugated/total bilirubin ratio is higher than 0.2, or the plasma conjugated bilirubin level is above 1.0 mg/dL. Significant amounts of delta-bilirubin are present only in prolonged cholestasis. Drug-induced hyperbilirubinemia is usually reversible and benign without underlying serious liver pathology (hepatocellular necrosis, cholestasis).

## 3. Metabolism and Transport of Bilirubin

**Figure 2:**
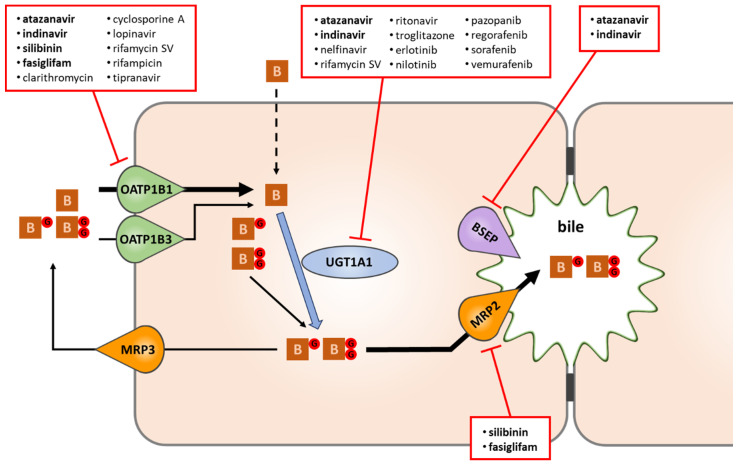
The fate of bilirubin in liver cells, and selected drugs causing hyperbilirubinemia via interference with bilirubin disposition. Of the drugs known to induce clinically relevant hyperbilirubinemia, atanazavir, indinavir, silibinin, and fasiglifam (highlighted in bold) were found to interrupt bilirubin disposition at multiple steps (uptake, conjugation, bile formation). Some hyperbilirubinemia-inducing drugs interfere predominantly with OATP1B1-mediated bilirubin uptake (esp. those with IC_50_ ≤ 6 μM towards OATP1B1), while a subset of tyrosine kinase inhibitors with C_max_/IC_50_ > 1 and/or IC_50_ ≤ 12 μM yield a similar effect by potently blocking UGT1A1. B: bilirubin; BG and BGG: bilirubin mono- and bisglucuronide.

The main metabolic and transport pathways of bilirubin, along with pharmaceuticals acting as potent inhibitors of each process, are shown in Figure 2. Unconjugated bilirubin is taken up into liver cells either by passive diffusion or via OATP1B1 and OATP1B3. Once in the hepatocytes, bilirubin is conjugated by UGT1A1. Most of the conjugated bilirubin is excreted into bile by the canalicular pump MRP2, while a smaller fraction is transported back to the blood through the sinusoidal membrane domain by MRP3. Interestingly, cyclic intraday variation in the serum levels of unconjugated bilirubin has recently been discovered in mice and linked to the regulation of UGT1A1 and MRP2 expression by the circadian clock [6]. Continuous bile flow, primarily fed by the canalicular bile salt export pump BSEP, is another prerequisite for normal biliary bilirubin clearance.

### 3.1. UGT1A1

UGT1A1 is a key Phase II enzyme that glucuronidates unconjugated bilirubin to mono- and bisglucuronides. UGT1A1 also metabolizes hormones and drugs, and polymorphic variants of the enzyme are accountable for adverse drug reactions [7]. In a genome-wide association study, the top variants in UGT1A1 explained about 18% of population-level variation in total bilirubin levels [8]. Mutations of this enzyme are responsible for Gilbert’s syndrome (GS) as well as Crigler-Najjar syndrome Type I and Type II [9]. In GS, reduced (20–30% of wild-type) glucuronidation activity of UGT1A1 causes lifelong mild unconjugated hyperbilirubinemia. The condition is common, affecting at least 5% of the Caucasian population, and essentially benign, although dose adjustment of certain UGT1A1 substrate drugs, especially in the field of oncology [10], may be necessary. Crigler-Najjar syndrome Type II is similarly benign and much less frequent compared to GS. Gilbert’s syndrome and Crigler-Najjar syndrome Type I also share a similar genetic background: in both conditions, missense mutations affecting the coding region as well as promoter TATA box TA repeat variants that reduce UGT1A1 production were identified. The UGT1A1*28 allele is a common example of hypomorphic promoter repeat variation, with 7 TA repeats instead of the wild-type 6 [11]. Crigler-Najjar syndrome Type I, on the other hand, is also rare but severe, often progressing to kernicterus within days after birth [12]. In Crigler-Najjar syndrome Type I, deleterious genetic lesions totally eliminate UGT1A1 activity and lead to potentially lethal hyperbilirubinemia [13]. Deletion of the Ugt1 locus from mice leads to neonatal lethality [14], whereas Gunn rats [15] carrying a spontaneous truncating mutation in the Ugt1a1 gene exhibit unconjugated hyperbilirubinemia and jaundice but are otherwise viable. Gunn rats, if left untreated, develop bilirubin encephalopathy and thus provide an excellent model for testing experimental therapies against Crigler-Najjar syndrome Type I (e.g., [16]).

### 3.2. OATP1B1/SLCO1B1 and OATP1B3/SLCO1B3

The role of OATP1B1 and OATP1B3 in the hepatic uptake of unconjugated and conjugated bilirubin has been confirmed in multiple in vitro, preclinical and clinical studies [17]. OATP1B1- and OATP1B3-mediated transport of unconjugated bilirubin was shown first in vitro [18,19]; subsequent clinical and drug inhibition studies have confirmed the in vivo role of OATP1B transporters in hepatic bilirubin uptake [20]. The contribution of OATP1B1 and OATP1B3 to the cellular uptake of conjugated bilirubin was also demonstrated first in an overexpressing HEK293 system [19], and later in vivo in transgenic mice [21]. In humans, concomitant pathogenic mutations of both the *SLCO1B1* and *SLCO1B3* genes cause Rotor syndrome [21] which presents, among other symptoms, with conjugated hyperbilirubinemia. Expression of OATP1B1 and 1B3 was found to be decreased in advanced liver diseases and correlated inversely with serum bilirubin levels [22]. In a genome-wide association study, the top variants of OATP1B1 explained about 1% of variation in total bilirubin levels, and this association remained highly significant after adjusting for individual UGT1A1 genotypes [8]. The *rs4149056* variant of the *SLCO1B1* gene was recently found to be associated with the risk of neonatal hyperbilirubinemia [23].

In adult humans, the plasma levels of unbound, unconjugated bilirubin are thought to be significantly lower than the K_m_ of OATP1B1 towards unconjugated bilirubin (7.6 nM). Therefore, OATP1B1-mediated transport of unconjugated bilirubin is unlikely to be saturated [24].

Both OATP1Bs are broad substrate specificity transporters with many drugs among their substrates [25], and drug–drug interaction (DDI) studies have revealed numerous instances of drug-mediated OATP1B inhibition [26]. Although OATP1B1 and OATP1B3 display very similar substrate and inhibitor specificities, drug inhibition studies have established a more decisive role for OATP1B1 in the transport of unconjugated bilirubin [24,27]. The 8-fold higher intrinsic clearance of OATP1B1-mediated versus OATP1B3-mediated transport of unconjugated bilirubin offers a feasible explanation for the difference.

### 3.3. MRP2/ABCC2

MRP2 is the efflux pump solely responsible for the canalicular excretion of glucuronidated, but not of unconjugated, bilirubin [28,29,30,31]. Intriguingly, apart from its canonical localization in the canalicular membrane domain, MRP2 was also detected in the nuclear envelope and perinuclear endoplasmic reticulum of hepatocytes where, in cooperation with co-localized conjugating enzymes, it may provide additional protection to the nucleus by extruding potentially genotoxic conjugates, and may regulate intranuclear concentrations of nuclear receptor ligands [32].

Genetic impairment of Mrp2/MRP2 function in humans (Dubin-Johnson syndrome) [33], rats (TR− rats; Eisai hyperbilirubinemic rats), and mice results in predominantly conjugated hyperbilirubinemia [21,34]. In Dubin-Johnson syndrome, the hereditary malfunction of MRP2 is compensated by overexpression of the sinusoidal efflux pump MRP3 [35], and patients characteristically show elevated serum levels of conjugated bilirubin [31].

Some drugs including silibinin [36,37], fasiglifam (TAK-875) [38], octreotide [39], and others [40] have been shown to inhibit MRP2 along with other hepatic bilirubin transporters, especially OATPs. However, no drug is known to cause hyperbilirubinemia through selective inhibition of MRP2 alone, and studies on larger datasets failed to confirm a major role for MRP2 in unconjugated hyperbilirubinemia [27].

### 3.4. MRP3/ABCC3

Human MRP3 is known to transport bilirubin glucuronides [41]. MRP3 is thought to play a key role in the homeostasis of conjugated bilirubin species as it effluxes bilirubin glucuronides into the blood from hepatocytes in the periportal zone for uptake through OATP1B1 and OATP1B3 by downstream hepatocytes in the centrilobular zone, a mechanism called “hepatocyte hopping” [21]. Several studies have addressed the role of Mrp3 in bilirubin homeostasis in rodents. Abcc3/Mrp3 knockout mice display lower serum levels of bilirubin glucuronides [42], and knocking out Mrp3 function compensates for increased serum bilirubin glucuronides in *Slco1b* knockout mice [21]. Nevertheless, altered bilirubin disposition due to disrupted MRP3 function has never been demonstrated directly in humans. The only study that attempted to link MRP3 mutations/variations to bilirubin homeostasis in humans focused on the natural c.3890G>A variant that introduces a conservative amino acid change (p.R1297H). This mutant, however, was at least as efficient as the wild type at transporting bilirubin glucuronides [41].

Dual inhibition of MRP2 and MRP3 can be logically predicted to increase bilirubin levels in human hepatocytes, as both proteins transport conjugated bilirubin and MRP3 serves as a relief transporter for MRP2. The glucuronide conjugate of fasiglifam was shown to potently inhibit both MRP2 and MRP3, and fasiglifam acyl glucuronide increased intrahepatocytic bilirubin levels 9-fold in rats [43]. In humans, systematic drug inhibition studies to elucidate the effect of MRP3 inhibition on tissue bilirubin levels and/or toxicity are still to be done.

### 3.5. BSEP

BSEP is the main canalicular transporter for monovalent bile acids, and although it is not directly involved in bilirubin disposition, its malfunction or blockade disrupts normal bile flow which is a prerequisite to adequate bilirubin clearance. Thus, cholestasis caused by aberrant or absent BSEP function may be accompanied by high bilirubin levels [27,44]. In Progressive Familial Intrahepatic Cholestasis Type 2 (PFIC2), the genetic dysfunction of BSEP manifests in early-onset jaundice due to elevated plasma levels of conjugated bilirubin [45]. Cholestasis and conjugated hyperbilirubinemia that often accompany chronic treatment with fusidic acid are thought to arise from concomitant inhibition of OATPs and BSEP by this antimicrobial agent [46].

## 4. The Toxicity of Bilirubin

In the physiological concentration range, unconjugated bilirubin is a potent antioxidant [47], and even chronic mild elevation of serum bilirubin—such as in Gilbert’s syndrome—is a largely innocuous condition that may suppress inflammation [48] and protect against cardiovascular disease [49,50]. However, toxic effects take over at high concentrations, especially in neonates. High unconjugated bilirubin levels in neonates who experience excessive hemolysis increase DNA damage and oxidative stress systemically [51]. The developing nervous system is particularly vulnerable to high levels of unconjugated bilirubin, and neonatal hyperbilirubinemia may lead to kernicterus, a severe neurotoxic injury characterized by auditory impairment as well as locomotory and ocular movement disorders [52]. Excess bilirubin in the central nervous system arrests cell divisions, provokes apoptosis of neurons, astrocytes and oligodendrocytes, triggers the release of inflammatory cytokines, disturbs synaptic potentials, and interferes with neurotransmission [47,52,53,54,55]. Bilirubin was also shown to interact deleteriously with multiple components of the ubiquitin-proteasome system; thus, neurotoxicity may at least in part be attributed to impaired proteasome function and inhibition of deubiquitination activity [56]. Bilirubin may disrupt other generic vital functions common to all cell types such as mitochondrial oxidative phosphorylation, maintenance of DNA stability, and protein synthesis [1].

In adults, hyperbilirubinemia presenting with jaundice is typically not regarded as a health hazard per se, rather as an indicator of disrupted bilirubin homeostasis due to hemolysis or hepatic malfunction. However, animal models suggest that unconjugated hyperbilirubinemia may be directly damaging to adults as well, e.g., via stimulating platelet apoptosis and thereby causing thrombocytopenia [57].

## 5. In Vitro Testing of Bilirubin Metabolism and Transport

Well-established in vitro assays exist for testing the potential interference of a chemical entity with UGT1A1, OATP1B1, OATP1B3, BSEP, MRP2, or MRP3 function. Due to the unfavorable stability and solubility properties of bilirubin and its glucuronides, in vitro assays tend to employ substrates that provide a wider dynamic range. For transport studies, radiolabeled substrates are usually preferred over unlabeled substrates. Transport assays typically utilize overexpressing systems based on cell lines like HEK293, while sandwich-cultured hepatocytes [58] are the model of choice for a more comprehensive assessment of bilirubin homeostasis.

UGT1A1 is usually tested in human liver microsomes with β-estradiol as a probe substrate [27,59] and the amount of estradiol-3-glucuronide is determined by liquid chromatography-tandem mass-spectrometry (LC-MS/MS) [60]. UGT1A1-containing microsomes can also be prepared from human cell lines [61] or baculovirus-infected insect cells overexpressing human UGT1A1 [62], and bilirubin [63] or etoposide [64] can be used as alternative substrates in inhibition studies.

OATP1B1 and OATP1B3 inhibition is mostly assessed using stably transfected or transduced cells and estradiol-17β-glucuronide as a substrate [24,59], with statins also employed in some studies [27]. Albeit tritiated bilirubin is available, the low dynamic range of the assay advocates against its use in inhibition assays [59]. In high-throughput screening (HTS) studies, sodium-fluorescein can be used as a fluorescent probe [65]. Although OATP1B1 and OATP1B3 have broadly overlapping substrate specificities, if inhibition needs to be evaluated in cells co-expressing both transporters estrone-3-sulfate and cholecystokinin-octapeptide (CCK8) can be used as substrates with marked preference for OATP1B1 and OATP1B3, respectively [66].

MRP2 and MRP3 preferably transport glucuronides; therefore, estradiol-17β-glucuronide is an obvious choice of substrate for MRP2/MRP3 vesicular transport assays [43,67]. However, since the in vitro kinetics of MRP2-mediated estradiol-17β-glucuronide transport is complex and cannot be described with a simple Michaelis-Menten model, some studies have employed other substrates such as the fluorescent 5(6)-carboxy-2′,7′-dichlorofluorescein (CDCF) [68,69].

Tritiated taurocholate is used almost exclusively as the probe substrate in BSEP inhibition assays. Taurocholate, however, is of limited relevance to humans as it is neither the most abundant nor the most toxic bile salt in our species [70]. Glycochenodeoxycholate (GCDC), the most abundant bile salt in humans, should therefore be preferred as a probe substrate if clinical relevance is paramount. The kinetics of BSEP-mediated GCDC transport has been recently characterized [71].

## 6. Prediction of Drug-Induced Hyperbilirubinemia

Hyperbilirubinemia is a common side effect associated with many different classes of clinical drugs [72] including Hepatitis C antivirals [73], antibiotics [74], and various anticancer agents [7,75]. The mechanism of drug-induced unconjugated hyperbilirubinemia often involves inhibition of the key transporters and enzymes of the bilirubin disposition machinery. Most studies on drug-induced unconjugated hyperbilirubinemia have focused on the inhibition of OATP1B1, OATP1B3, UGT1A1 and MRP2, with MRP3 and BSEP also included in some assessments. Table 1 summarizes clinical and in vitro data on drugs associated with, or suspected to cause, unconjugated hyperbilirubinemia. Of the nine drugs assessed in multiple studies—atazanavir, cyclosporine A, indinavir, nelfinavir, rifamycin SV, rifampicin, ritonavir, silibinin, and saquinavir—five (atazanavir, cyclosporine A, indinavir, rifamycin SV, rifampicin) were unequivocally confirmed to cause hyperbilirubinemia. Nelfinavir and ritonavir were classified discordantly by different studies as either causative or non-causative, possibly due to differences in the criteria applied. Additionally, ritonavir is often co-administered with other antiviral drugs, which may obscure its potential contribution to hyperbilirubinemia. Saquinavir, despite being a potent in vitro inhibitor of OATP1Bs, was non-causative alone but causative in combination with ritonavir. Silibinin at normal therapeutic doses did not cause any adverse events in healthy volunteers [76]; however, when applied at high doses [77] and/or administered to vulnerable populations like patients with chronic liver disease [36,37,78], hyperbilirubinemia was a frequently reported side effect.

Various readouts, clarified in Table 2, have been used to link clinical hyperbilirubinemia with transporter and/or enzyme inhibition. The simplest readout was the IC_50_ value. C_max_/IC_50_ and unbound C_max_/IC_50_ (f_u_·C_max_/IC_50_) values have also been utilized either alone or as part of the R = (1 + C_max_/IC_50_) or R_free_ = (1 + (f_u_·C_max_/IC_50_) formulas. The intrinsic F_i_ value [24] that indicates the inhibitable fraction of membrane transport clearance is also based on IC_50_ and unbound C_max_ values and is calculated as F_i_ = 1- [IC_50_/(IC_50_ + f_u_·C_max_)]. Finally, R values based on unbound liver inlet concentration such as R_in,free_ = (1 + [f_u_·I_in,max_/IC_50_]) were calculated in several studies.

The most widely tested transporter was OATP1B1 (usually along with OATP1B3), and cut-offs based on OATP1B1 inhibition were unanimously accepted as useful predictors of hyperbilirubinemia. In the study by Nakakariya et al. [79], the cut-off of IC_50_ ≤ 6 µM for OATP1B1 inhibition reliably identified compounds that cause hyperbilirubinemia at a frequency of ≥1%, although it was not specified whether these compounds induce accumulation of unconjugated or conjugated bilirubin. OATP1B1 inhibition also indicated hyperbilirubinemia when using the R_free_ > 1.1 [27], F_i_ > 0.2 [24,59], or R_in,free_ ≥ 1.5 [59,79] cut-offs. Therefore, OATP1B1 inhibition is clearly of great value in assessing the risk of drug-induced hyperbilirubinemia.

Of note, only the IC_50_ values but no other predictor indicated hyperbilirubinemia risk for fasiglifam, an investigational G protein-coupled receptor 40 agonist terminated late in phase III due to liver safety concerns [81]. Predictions based on F_i_ and R_in,free_ may be compromised by the high protein binding (f_u_ < 0.2%) of fasiglifam, which may lead to systematic underestimation of its effective inhibitory potential. The same may hold true for saquinavir with low IC_50_ values against OATP1Bs but an unbound fraction of only ~2%.

Despite the broad overlap between the substrate and inhibitor profiles of both OATP1B transporters, the correlation between OATP1B3 inhibition and hyperbilirubinemia is less compelling compared to OATP1B1. Given the lower expression and smaller contribution to intrinsic bilirubin clearance of OATP1B3 relative to OATP1B1, the inclusion of OATP1B3 inhibition data seems to be mostly redundant as long as OATP1B1 is being considered.

Interestingly, when the potential implication of OATP1B1/OATP1B3 inhibition in drug-induced hyperbilirubinemia was tested based on *in silico* predicted inhibitory potencies [82], no strong link between OATP1B inhibition and hyperbilirubinemia was revealed. The authors discuss methodical pitfalls that may confound a true correlation as well as possible physiological mechanisms that may explain the lack of it, but do not conclusively refute an existing association [83].

The predictive power of BSEP and MRP2 inhibition was evaluated in only one study [27] which concluded that the R_free_ value of >1.1 for BSEP inhibition, in combination with a similar cut-off for OATP1B1 and UGT1A1 inhibition, could be useful in predicting hyperbilirubinemia. Although some drugs included in this study, as well as fasiglifam and silibinin, display comparatively potent inhibition of MRP2 in vitro, no cut-off for MRP2 inhibition predictive to hyperbilirubinemia has been suggested so far, and isolated inhibition of MRP2 without affectedness of other hepatic bilirubin transporters is unlikely to cause clinically relevant unconjugated hyperbilirubinemia.

Although UGT1A1 inhibition data also leave room for some controversy, UGT1A1 was confirmed in multiple studies as an independent and significant contributor to unconjugated hyperbilirubinemia. In the study of Chang et al. [27], the R_free_ > 1.1 cut-off, along with a similar cut-off for OATP1B1 and BSEP, was helpful in separating the hyperbilirubinemic compounds atazanavir and indinavir from nelfinavir and ritonavir as well as the cholestatic compounds bromfenac, troglitazone and trovafloxacin, all considered non-hyperbilirubinemic. Qosa et al. tested a set of tyrosine kinase inhibitors (TKIs) for UGT1A1 inhibition [75], and they found that C_max_/IC_50_ ratios greater than 1 were associated with hyperbilirubinemia: erlotinib, nilotinib, regorafenib, pazopanib, sorafenib and vemurafenib with C_max_/IC_50_ > 1 all had high Empirical Bayesian Geometric Mean (EBGM) scores for this condition (Table 3). In this study, F_i_ and R_In,free_ also correlated well with EBGM scores, while in the study of Chiou et al. [59] neither F_i_ nor R_in,free_ data for UGT1A1 provided a clinically meaningful cut-off. In our assessment of the TKI data from Qosa et al. [75], IC_50_ values themselves showed a better correlation with EBGM scores than C_max_/IC_50_ ratios: all compounds but dasatinib with an IC_50_ ≤ 12 µM had an EBGM score >2. Based on this cut-off, dasatinib was the only false positive as it had an IC_50_ of 9 µM but a borderline EBGM score of 1.913, and sunitinib was the only false negative with an EBGM score of 2.955 and an IC_50_ of 131 µM. Atazanavir and rifamycin SV, the two compounds in the study by Chiou et al. that had an F_i_ > 0.2 and R_in,free_ ≥ 1.5 for UGT1A1, also had IC_50_ values ≤ 12 µM. In the study of Chang et al. [27], in addition to atazanavir and indinavir that were classified as causative agents of hyperbilirubinemia, ritonavir, nelfinavir, and troglitazone also had UGT1A1 IC_50_ values ≤12 µM. Although ritonavir and nelfinavir were considered as non-hyperbilirubinemic in this particular study, they were both implicated in hyperbilirubinemia by Nakakariya et al. [79]. Thus, the UGT1A1 inhibition cut-off derived from the TKI study probably remains valid in a broader context.

For the somatostatin analog octreotide, hyperbilirubinemia would not be expected based on any single IC_50_ value or calculated predictor, which is at odds with occasional reports of elevated serum bilirubin upon octreotide treatment [39]. Such false negative predictions draw attention to the fact that simple risk assessment models may be unable to account for the interaction of multiple factors, e.g., simultaneous inhibition of multiple hepatic bilirubin transporters (as well as potentially other, as yet unidentified, targets) as is the case with octreotide.

## 7. Conclusions

Unconjugated hyperbilirubinemia has been linked most consistently to the function of OATP1B1 and UGT1A1. OATP1B1 inhibition was commonly assessed in screening-type in vitro studies. For compounds in the discovery phase, the OATP1B1 IC_50_ ≤ 6 µM cut-off is to be considered as a predictor that can be calculated without a knowledge of the actual or calculated C_max_. Predictors that require more complex testing and computation of data can be used for drug candidates in late discovery and at clinical Phase I-II. For OATP1B1, the R_in,free_ = 1 + (f_u_ * [I_in,max_/IC_50_) ≥ 1.5 cut-off appears to be the consensus emerging from multiple studies. It is of note that DDI calculations for OATP1B1 also employ the unbound inlet concentration. For UGT1A1, based on available evidence IC_50_ ≤ 12 µM can be suggested as a provisional cut-off, but further studies are warranted to validate this value.

## Figures and Tables

**Table 1:** Correlation of clinically observed hyperbilirubinemia with in vitro transporter/enzyme inhibition.

Drug	Hyperbilirubinemia—Clinical Data	Cholestasis—Dlinical Data	OATP1B1		OATP1B3		BSEP		MRP2		UGT1A1		Reference
			K_i_ or IC_50_ (µM)	Calculated Predictor(s)	IC_50_ (µM)	Calculated Predictor(s)	IC_50_ (µM)	Calculated Predictor(s)	IC_50_ (µM)	Calculated Predictor(s)	IC_50_ (µM)	Calculated Predictor(s)	
Amprenavir	no		7.5	F_i_ = 0.19; R_in,free_ = 1.3–1.6	38	F_i_ = 0.04; R_in,free_ = 1.1					64	F_i_ = 0.027; R_in,free_ = 1.0 - 1.1	[59]
Atazanavir	yes		**0.9**	**R_free_ = 1.6**	**3.7**	R_free_ = 1.1	3.1	**R_free_ = 1.2**	>100		**6.8**	**R_free_ = 1.4**	[27]
Atazanavir	yes		**0.37**	**F_i_ = 0.74; R_in,free_ = 4.2 - 5.6**	**0.82**	**F_i_ = 0.56; R_in,free_ = 2.5 - 3.0**					**0.76**	**F_i_ = 0.58; R_in,free_ = 2.6 - 3.2**	[59]
Bromfenac		yes	>100		>100						77	R_free_ = 1.0	[27]
Clarithromycin	yes, more than 1%		**5.1**	**R_in,free_ = 6.2**	9.8	**R_in,free_ = 3.7**	>100		>100				[79]
Cyclosporine A	yes, some patients		**0.2**	**F_i_ = 0.43**									[24]
Cyclosporine A	yes, more than 1%		**0.55**	**R_in,free_ = 3.8**	**0.5**	**R_in,free_ = 4.1**							[79]
Cyclosporine A	yes		**0.13**	**F_i_ = 0.54; R_in,free_ = 4.9**	0.057	**F_i_ = 0.73; R_in,free_ = 9.6**					59	F_i_ = 0.025; R_in,free_ = 1.0	[59]
Fasiglifam (TAK-875)	yes	yes	**2.28**	F_i_ = 0.003; R_in,free_ = 1.0	**3.98**	F_i_ = 0.002; R_in,free_ = 1.0			2.41	F_i_ = 0.003; R_in,free_ = 1.0			[38,80]
Indinavir	yes, some patients		6.8	**F_i_ = 0.41**									[24]
Indinavir	yes		**4.1**	**R_free_ = 1.7**	>100		3.1	**R_free_ = 1.9**	>100		**6.8**	**R_free_ = 1.4**	[27]
Indinavir	yes		8.3	**F_i_ = 0.38; R_in,free_ = 2.0 - 3.2**	16	**F_i_ = 0.24; R_in,free_ = 1.5 - 2.1**					35	F_i_ = 0.013; R_in,free_ = 1.2 - 1.5	[59]
Lopinavir	yes, more than 1% ^1^		1		6,7								[79]
Lopinavir/ritonavir	yes, more than 1%			**R_in,free_^2^ = 2.0**		**R_in,free_^2^ = 1.2**							[79]
Nelfinavir	yes, more than 1%		5.3	**R_in,free_ = 1.5**	15	**R_in,free_ = 1.2**							[79]
Nelfinavir	no		2	R_free_ = 1.0	>100		24	R_free_ = 1.0	>100		4.8	R_free_ = 1.0	[27]
Octreotide	yes, some patients		23	R_in,free_ = 1.0	68	R_in,free_ = 1.0			116.6	R_in,free_ = 1.0			[39]
Rifamycin SV	yes, all patients		0.2	**F_i_ = 0.96**									[24]
Rifamycin SV	yes		0.05	**F_i_ = 0.99; R_in,free_ = 104 - 126**	0.052	**F_i_ = 0.99; R_in,free_ = 104 - 122**					**12**	F_i_ = 0.29; R_in,free_ = 1.4 - 1.5	[59]
Rifampicin	yes, more than 1%		1.3	**R_in,free_ = 12**	1.5	**R_in,free_ = 11**							[79]
Rifampicin	yes		0.59	**F_i_ = 0.70; R_in,free_ = 4.6**	0.22	**F_i_ = 0.86; R_in,free_ = 10.7**					33	F_i_ = 0.04; R_in,free_ = 1.1	[59]
Ritonavir	yes, more than 1% ^1^		2.5		7.6								[79]
Ritonavir/saquinavir	yes, more than 1%			**R_in,free_^2^ = 1.4**		**R_in,free_^2^ = 1.2**							[79]
Ritonavir	no		0.5	**R_free_ = 1.2**	>100		2.6	R_free_ = 1.0	>100		**3.1**	R_free_ = 1.0	[27]
Saquinavir	no		1.2	F_i_ = 0.07									[24]
Saquinavir	no, less than 1% ^1^		6.1		57								[79]
Saquinavir	no		**0.41**	F_i_ = 0.021; R_in,free_ = 1.1	**0.47**	F_i_ = 0.020; R_in,free_ = 1.1					23	F_i_ = 0.00038; R_in,free_ = 1.0	[59]
Silibinin ^3^	yes, in HCV patients [36] and/or at high doses [77]		9.7/8.5	R_in,free_ = 1.02/1.08	**2.7/5.0**	R_in,free_ = 1.08/1.14							[37]
Silibinin ^4^			**3.28**	**F_i_ = 0.31;** **R_free_ = 1.46**	**5.0**	**F_i_ = 0.23;** **R_free_ = 1.30**	>100		6.79	F_i_ = 0.18; R_free_ = 1.22			[36]
Tipranavir	yes, more than 1%1		0.7		0.61								[79]
Tipranavir/ritonavir	yes, more than 1%			**R_in,free_^2^ = 3.6**		**R_in,free_^2^ = 3.9**							[79]
Troglitazone		yes	**1.2**	R_free_ = 1.0	>100		18	R_free_ = 1.0	17	R_free_ = 1.0	4.5	R_free_ = 1.0	[27]
Trovafloxacin		yes	>100		>100		1.7	**R_free_ = 1.9**	>100		>100		[27]

^1^ Co-treatment assumed. ^2^ Cumulative value. ^3^ Calculation for 700 mg silymarin/day (116 mg silybin A + 160 mg silybin B); IC_50_ and R_in,free_ values are shown separately for silybin A/silybin B. ^4^ Calculation for high-dose treatment (20 mg/kg body weight/day silibinin); IC_50_ and R_free_ values are for total silibinin. Data discussed herein and considered relevant based on cut-off values (IC_50_, F_i_, R_free_, or R_in,free_ for OATP1B1 and IC_50_ for UGT1A1) suggested by the original articles or by this review are depicted in bold.

**Table 2:** Definitions of predictors calculated based on in vitro inhibition data

Calculated Predictor	Formula
R	$1+\frac{C_{max}}{IC_{50}}$
R_free_	$1+f_{u}*\;\frac{C_{max}}{IC_{50}}$
R_in,free_	$1+f_{u}*\;\frac{I_{in,max}}{IC_{50}}$
F_i_	$1-\frac{IC_{50}}{IC_{50}+f_{u}*\;C_{max}\;}$

C_max_: maximum serum concentration; f_u_: unbound fraction; I_in,max_: maximum liver inlet concentration.

**Table 3:** Correlation of clinically observed hyperbilirubinemia with in vitro inhibition of UGT1A1 for a set of tyrosine kinase inhibitors

Drug	EBGM Score for Hyperbilirubinemia	C_max,total_ (μM)	IC_50_ (µM)	IC_50_ ≤ 12 µM	C_max,total_/IC_50_	(C_max,total_/IC_50_) > 1	In vivo F_i_	R_in,free_
Afatinib	1.859	0.099	13.4	TN	0.0074	TN	0.0004	1.006
Axitinib	**2.262**	0.072	**9.9**	TP	0.0073	FN	0.0001	1.0002
Bosutinib	0.668	0.404	74.6	TN	0.0054	TN	0.0002	1.0043
Cabozantinib	0.586	0.218	82.1	TN	0.0026	TN	0.0001	1.0007
Cediranib	–	1.292	–	–	–	–	–	–
Ceritinib	1.418	2.186	35.2	TN	0.0621	TN	0.0056	1.0228
Crizotinib	–	0.913	99.5	TN	0.0092	TN	0.0004	1.0023
Dasatinib	1.913	0.163	**9**	*FP*	0.0182	TN	0.0013	1.0164
Erlotinib	**3.07**	3.410	**1.6**	TP	**2.0743**	TP	0.094	1.243
Imatinib	1.73	7.716	130	TN	0.0589	TN	0.0006	1.003
Lapatinib	**15.073**	2.943	**5.2**	TP	0.6714	*FN*	0.0132	1.1116
Nilotinib	**8.742**	2.571	**1.1**	TP	**2.3232**	TP	0.0444	1.149
Nintedanib	1.177	0.064	–	–	–	–	–	–
Pazopanib	**2.631**	5.240	**1.9**	TP	**2.8167**	TP	0.0274	1.0695
Ponatinib	**2.252**	0.156	**11.1**	TP	0.014	*FN*	0.0001	1.0011
Regorafenib	**13.602**	8.278	**1**	TP	**8.1545**	TP	0.0081	1.1358
Sorafenib	**5.681**	10.54	**2.7**	TP	**3.8858**	TP	0.1627	1.3497
Sunitinib	**2.955**	0.231	131	*FN*	0.0018	*FN*	0.0002	1.0021
Vandetanib	1.283	4.261	98.3	TN	0.0433	TN	0.0004	1.0017
Vemurafenib	**3.368**	125.3	**10.9**	TP	**11.4531**	TP	**0.3641**	**1.6622**

Data from Qosa et al. [75] TN: true negative, TP: true positive, *FN*: false negative, *FP*: false positive. Positive results are in bold.
